# A Microsurgical Technique for Removing the Spermatheca of Bumblebee Females and Its Application

**DOI:** 10.3390/insects16070734

**Published:** 2025-07-18

**Authors:** Mingsheng Zhuang, Fan Yang, Zhongyan Xia, Yu Fei, Fugang Liu, Zhengyi Zhang, Zhihao Zhang, Jilian Li

**Affiliations:** State Key Laboratory of Resource Insects, Institute of Apiculture Research, Chinese Academy of Agricultural Sciences, Beijing 100093, China; yueju0208@126.com (M.Z.); yangfan920302@163.com (F.Y.); xzy202992@163.com (Z.X.); 19970035818@163.com (Y.F.); lfg125998@163.com (F.L.); zzy472133358@163.com (Z.Z.); 18661642791@163.com (Z.Z.)

**Keywords:** *Bombus terrestris*, bumblebee, spermatheca, social insect, a microsurgical technique

## Abstract

To solve the technical bottleneck caused by the absence of a feasible method for removing the spermatheca in social insects, we developed a microsurgical technique specifically designed for bumblebee females. In this study, the invention of this technique is based on the anatomical characteristics of the sting chamber of bumblebees and uses a bespoke scalpel to precisely remove the spermatheca, which is small in size and deeply embedded within the body. During the removal operation, a small wound was observed and a small amount of hemolymph flowed out. The wound healed very quickly and the survival rate of treated individuals was high. The results showed that there was no significant impact on the critical life activities of queens and workers, including longevity, mating behavior, oviposition capacity, and overwintering survival rate after the spermatheca was removed using this technique. These findings further confirm the feasibility and applicability of the technique and provide strong technical support for exploring the evolutionary dynamics and potential function of the spermatheca in social insects.

## Simple Summary

In this study, we developed a microsurgical technique for removing the spermatheca of bumblebee females. Using this technique, we surgically removed the spermathecae of *Bombus terrestris* females (queens and workers) and investigated the effects on their key life activities, including mating behavior, longevity, the survival rate of females overwintering, and oviposition. The results showed that there was no significant impact on the mating behavior, longevity, or oviposition of queens and workers after their spermathecae were removed. These findings confirm the feasibility and practicality of this technique and provide strong technical support for elucidating the function of the spermatheca and the mechanisms of its degeneration.

## 1. Introduction

The internal organs of insects play critical roles in their survival, development, reproduction, behavioral regulation, and ecological adaptation [1,2,3]. Therefore, the study of their structure and physiological functions holds considerable practical significance for the conservation and utilization of insects. Organ ablation is a traditional method used to investigate the physiological functions of these organs [4,5]. As early as 1922, the experiments were conducted to verify the functions of specific organs in aspects such as developmental regulation, behavioral control, and hormone synthesis by removing these organs [6]. For example, the removal of testes, seminal vesicles, and accessory glands in locusts was found to disrupt the synthesis of juvenile hormone [7]. Ablation of the corpora allata in honeybees demonstrated that this gland plays a role in the secretion of juvenile hormone and in regulating worker behavior [1,2]. Similarly, it was confirmed through the removal of the mandibular glands of the bumblebee queen that the secretions from these glands can serve as chemical signals to regulate the development of the larvae [8].

The spermatheca is a unique organ in insect females that stores sperm and is widely present in most of the taxonomic groups within the Insecta class. Its primary functions include receiving, storing, maintaining sperm viability, and enabling fertilization of the egg [9,10]. In social insects such as bees and ants, queens typically mate with drones only once but can rely on the spermatheca to store millions of sperm for long-term use, thereby sustaining efficient oviposition throughout their lifetime [11]. The methods for studying on the functions and composition of the spermatheca typically include tissue dissection, histological sectioning, and immunostaining [12,13,14]. Because this organ is extremely small and deeply embedded within the body, with a concealed location and closely connected to other organs, particularly in social insects such as bumblebees [15], at present, there is a lack of any method to precisely remove this organ, which has become the main technical bottleneck limiting the further study of its functions.

Reproduction is an important guarantee for the continuation of a species [16]. The reproduction of bumblebee queen requires several crucial stages, including mating, diapause (overwintering), and oviposition [17]. Bumblebees have been regarded as ideal model organisms due to their unique biological characteristics. On the one hand, our previous research revealed that bumblebee workers retain structurally intact spermathecae and exhibit queen-like reproductive traits, such as the ability to mate and found colonies [15,18]. On the other hand, *Bombus terrestris* has a relatively large body and a small spermatheca [19]. Moreover, in previous studies, we found that the spermatheca is easily exposed and identified within the sting chamber (unpublished data), which provides ideal conditions for conducting microsurgical operations.

Therefore, in this study, we used bumblebees as a model to develop a microsurgical technique for removing the spermatheca. The feasibility of the technique was validated by assessing its impact on the longevity and reproductive traits of queens and workers after their spermathecae were removed. The results showed that the removal of the spermatheca had no significant effect on their longevity, mating behavior, oviposition capacity, or overwintering survival in either caste. The feasibility of this technique provides strong technical support for further exploring whether the spermatheca has any potential functions other than sperm storage, and it also establishes an experimental foundation for exploring associated reproductive mechanisms.

## 2. Materials and Methods

### 2.1. Sampling Bumblebees for Trials

In this study, we used *Bombus terrestris* as a model species. Two hundred commercial colonies of *B. terrestris* were obtained from a commercial supplier based at the Institute of Apicultural Research, Chinese Academy of Agricultural Sciences. These colonies were reared in environmentally controlled rooms (temperature 28 ± 1 °C, relative humidity 60 ± 5%, in the dark), with 50% sucrose solution (*w*/*w*) and oilseed rape pollen provided ad libitum. Each test bee collected from these colonies was individually kept in a small plastic box (6 × 12 × 8 cm).

### 2.2. The Procedure for Removing the Spermatheca of Bumblebees

In this study, the callow females were used for all trials and anesthetized by carbon dioxide to prevent them from moving during the operation. The sting chamber of each test bee was gently opened using an artificial insemination instrument for bumblebees (VE-AIIOQB-H1.0-A, Shanghai Suosheng Biotechnology Co., Ltd., Shanghai, China), the spermatheca was exposed, the longitudinal axis of the bespoke scalpel for bumblebees (Chinese patent no. ZL202411634155.0) [20] was first aligned at an angle of approximately 35 degrees to the vertical, and the tip of the scalpel was inserted into the area of the spermatheca approximately 1 to 2 mm, so that it could fall into the groove of the scalpel. Then, we used another scalpel to cut the spermatheca tube and remove the entire spermatheca; the whole procedure is shown in Supplementary Video S1. The sting chamber of the female was photographed using a camera of the Bee Morphometer (MV-U210-V1.01-A, Shanghai Suosheng Biotechnology Co., Ltd., Shanghai, China) under a 20× light microscope.

### 2.3. Effects of the Removal of the Spermatheca on Female’s Longevity

To test the impact of the removal of their spermathecae on the longevity of bumblebee females, 90 newly-eclosed gynes were collected from 45 different colonies. In total, 90 callow workers were collected from 45 queen-right colonies (here, and throughout, we define queen-right colonies as comprising a queen and 30–40 workers without gynes or male larvae). Queens were randomly assigned to one of three treatment groups: the removal of the spermatheca group (QZC, n = 30), with the surgical removal of the spermatheca as described as described above; the sham surgery group (QSS, n = 30), which underwent the same procedure as the removal of spermatheca group but without actually removing the spermatheca; the control group (QCK, n = 30), without surgical treatment. Workers were also randomly assigned to one of three treatment groups: the removal of the spermatheca group (WZC, n = 30), the sham surgery group (WSS, n = 30), the control group (WCK, n = 30). After treatment, each bee was reared individually in a small plastic box, as described above. The survival number of bees and their survival duration (in days) were recorded.

### 2.4. Effects of the Removal of the Spermatheca on Female’s Mating Behavior

To test whether the removal of their spermatheca influences their mating behaviors, the gynes and callow workers were collected and randomly assigned to one of six treatment groups as described above. After treatment, each bee was reared individually in a small plastic box, as described above. We randomly chose females and exposed them to mating trials at five days post-eclosion. For each mating trial, the ratio of workers or gynes to males was 1:2, and the environmental conditions were as follows: the temperature was constant (25 ± 1 °C), and the size of the mating cages was 50 cm × 50 cm × 50 cm. All mating trials were repeated three times and the number of observed mating events and the duration of copulation (from initiation to separation) were recorded.

### 2.5. Effects of the Removal of the Spermatheca on Female’s Overwintering

To test whether the removal of their spermatheca influences their overwintering, the gynes and callow workers were collected and randomly assigned to one of six treatment groups as described above. After treatment, each bee was reared individually in a small plastic box, as described above. At 7 days, all bees were kept at 4 °C for the overwintering test. Queens were kept for 3 months. Given the lower survival capacity of workers in overwintering, workers were kept for 15 days. After the overwintering, all bees were returned to room temperature (23 °C) for 2 h and the number of survival bees were counted.

### 2.6. Effects of the Removal of Spermatheca on Female’s Oviposition

To test whether the removal of their spermatheca influences female oviposition, the gynes and callow workers were collected and randomly assigned to one of six treatment groups as described above. After treatment, each bee was reared individually in a small plastic box, as described above. To break the diapause of the females, we used carbon dioxide to anesthetize them for two successive days, with each anesthetization lasting for 10 min [21]. To stimulate females to lay eggs, each test individual was kept with two callow workers. These workers were replaced every 7 days to prevent them from laying eggs and thereby affecting the experimental results. All test bees were observed every day for 20 days. For each test group, the ovipositional number of each bee within five days and ovipositional rate of the test group were recorded.

### 2.7. Statistical Analysis

We used SPSS version 29 for analysis. Female longevity was assessed using the log-rank (Mantel–Cox) test. The survival and mating success rates of females at day 5 were analyzed using G-tests; Chi-square tests were applied to fixed-point frequency comparisons with balanced group sizes, so the overwintering survival rates and ovipositional rates were analyzed using Chi-square tests. The mating duration and ovipositional number of each bee were analyzed using either the Kruskal–Wallis test or one-way ANOVA, depending on the data distribution. If no significant difference is found, to evaluate the adequacy of the sample size and the risk of Type II error, a post hoc power analysis was performed using the lifelines package in Python 3.11.4.

## 3. Results

### 3.1. Morphological Changes in the Sting Chamber of Bumblebee After Removing the Spermatheca

**Figure 1:**
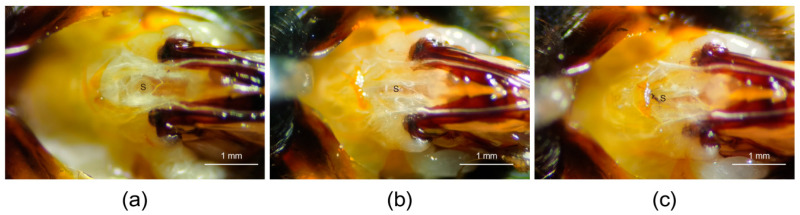
Morphological changes in the sting chamber of *B. terrestris* queens after the spermatheca was removed. (**a**) Sting chamber of a queen with an intact spermatheca, S = spermatheca. (**b**) Sting chamber after the spermatheca was removed, S = the wound site of post-removal. (**c**) Sting chamber 2 h after removal. S = scab at the wound site. The sting chamber of the queen was photographed using a camera of the Bee Morphometer (MV-U210-V1.01-A) under a 20× light microscope.

Due to the unique reproductive structure of bumblebees, its spermatheca can be clearly observed when the sting chamber is opened (Figure 1a). After the microsurgery is completed, the spermatheca not being observed in the sting chamber is used to determine whether the microsurgery was successful. A small wound can be observed and a small amount of hemolymph flows out during the operation (Figure 1b). However, due to the small size of the wound, a black scab is formed at the wound site within about 40 min (Figure 1c).

### 3.2. Effects of the Removal of the Spermatheca on Female’s Longevity

**Figure 2:**
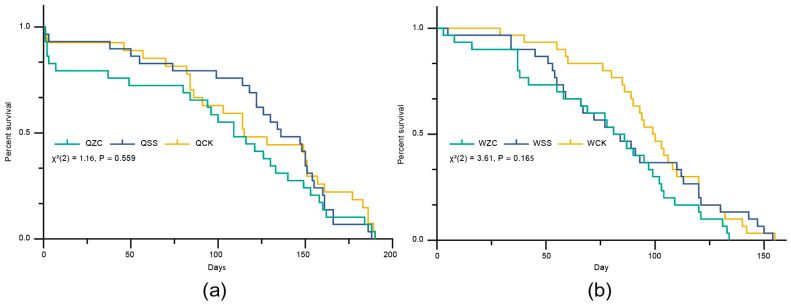
Effects of the removal of the spermatheca on *B. terrestris* females’ longevity. Q = queen, W = worker, ZC = spermatheca removal treatment, SS = sham-operated treatment, CK = untreated control. (**a**) Effects of the removal of the spermatheca on queens’ longevity (QZC: n = 30; QSS: n= 30; QCK: n = 30). (**b**) Effects of the removal of the spermatheca on workers’ longevity (WZC: n = 30; WSS: n = 30; WCK: n = 30). Data in (**a**,**b**) were analyzed using the log-rank (Mantel–Cox) test.

After 190 days, there were no significant differences in longevity among the queen treatment groups (QZC = 96.13 ± 63.72 days, QSS = 118.83 ± 54.48 days, QCK = 106.90 ± 63.48 days (mean  ±  SD); log-rank (Mantel–Cox) test: *χ*^2^(2) = 1.162, *p* = 0.559; Figure 2a). The estimated statistical power for this comparison was also low (0.478), and the possibility of a Type II error cannot be excluded. Similarly, there was no significant difference in longevity among the worker treatment groups (WZC = 77.10 ± 37.25 days, WSS = 86.27 ± 38.84 days, WCK = 98.03 ± 29.79 days (mean ± SD); log-rank (Mantel–Cox) test: *χ*^2^(2) = 3.608, *p* = 0.165; Figure 2b). The estimated statistical power for this comparison was also low (0.491), and the possibility of a Type II error cannot be ruled out.

Taken together, these results suggest that spermatheca removal or the sham operation did not cause a significant difference in the longevity of queens or workers. However, the relatively low statistical power indicates that further studies with larger sample sizes are necessary to confirm these findings.

### 3.3. Effects of the Removal of the Spermatheca on Female’s Mating Behavior

**Figure 3:**
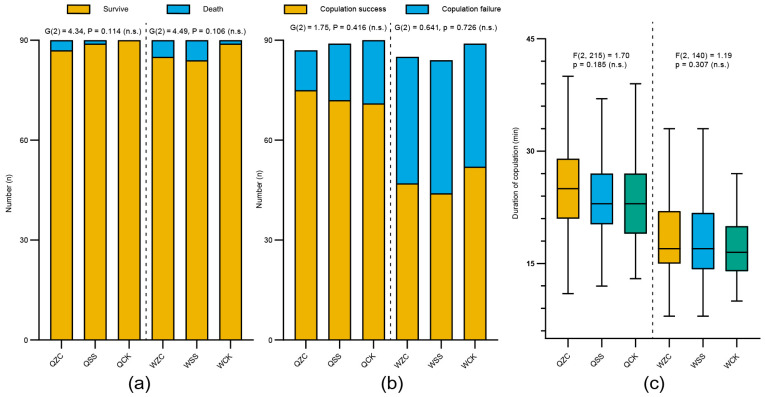
Effects of the removal of the spermatheca on *B. terrestris* females’ mating behavior. Q = queen; W = worker; ZC = removal treatment of spermatheca; SS = sham-operated treatment; CK = untreated control. (**a**) Survival rate on day 5 after the removal of the spermatheca (QZC: n = 90, QSS: n = 90, QCK: n = 90; WZC: n = 90, WSS: n = 90, WCK: n = 90). (**b**) All test bees were provided the opportunity to mate at five days post-removal and the mating success rate was recorded (QZC: n = 87, QSS: n = 89, QCK: n = 90; WZC: n = 85, WSS: n = 84, WCK: n = 89). (**c**) Copulation duration of treatment groups (from initiation to separation, QZC: n = 75, QSS: n = 72, QCK: n = 71; WZC: n = 47, WSS: n = 44, WCK: n = 52). Box plots consist of the box denoting the interquartile range (IQR), bound by the 25th and 75th percentiles, the median line shown within the box, and the whiskers representing the rest of the data distribution with outliers denoted by points greater than ±1.5 × IQR. Data in (**a**,**b**) were analyzed using G-tests. Data in (**c**) were analyzed using one-way ANOVA. Mating experiments were repeated three times, and as patterns were consistent, data were combined for analysis.

On the fifth day after their spermathecae were removed, there was no significant difference in the survival rates among treatment groups for either queens or workers (queens: QZC = 87/90 (96.7%), QSS = 89/90 (98.9%), QCK = 90/90 (100%); *G*(2)  =  4.34, *p* = 0.114; workers: WZC = 85/90 (94.4%), WSS = 84/90 (93.3%), WCK = 89/90 (98.9%); *G*(2)  =  4.49, *p* = 0.106; Figure 3a). Although there were differences in the number of successfully mated females among different groups, there were no significant differences (queens: QZC = 75/87 (86.2%), QSS = 72/89 (80.9%), QCK = 71/90 (78.9%); *G*(2) = 1.75, *p* = 0.416; workers: WZC = 47/85 (55.3%), WSS = 44/84 (52.4%), WCK = 52/89 (58.4%); *G*(2) = 0.641, *p* = 0.726; Figure 3b). The power analysis showed that the probability of a Type II error cannot be ruled out in either case (power < 0.4 for survival; power < 0.2 for mating).

Prior to statistical comparison, we conducted Shapiro–Wilk tests to assess the normality of copulation duration data (QZC: *W* = 0.987, *p* = 0.615; QSS: *W* = 0.973, *p* = 0.117; QCK: *W* = 0.97, *p* = 0.083; WZC: *W* = 0.966, *p* = 0.189; WSS: *W* = 0.961, *p* = 0.148; WCK: *W* = 0.97, *p* = 0.205). The results showed that the data from all experimental groups conformed to a normal distribution. Therefore, we used one-way ANOVA to compare copulation duration among groups. In addition, there were no significant differences in copulation duration (from the initiation of copulation to separation) among different treatment groups (queens: QZC = 25.11 ± 5.64 min, QSS = 23.85 ± 5.09 min, QCK = 23.48 ± 6.07 min (mean ± SD); *F*(2, 215) = 1.70, *p* = 0.185; workers: WZC = 18.19 ± 5.08 min, WSS = 18.14 ± 5.05 min, WCK = 16.92 ± 3.74 min (mean ± SD); *F*(2, 140) = 1.19, *p* = 0.307; Figure 3c). Power analysis showed that the probability of a Type II error was sufficiently low (power ≈ 1.0).

These results indicate that neither the removal surgery of spermatheca nor the sham operation significantly affected the survival rate of female individuals, mating success, or mating behavior during the short-term period.

### 3.4. Effects of the Removal of the Spermatheca on Female’s Overwintering

**Figure 4:**
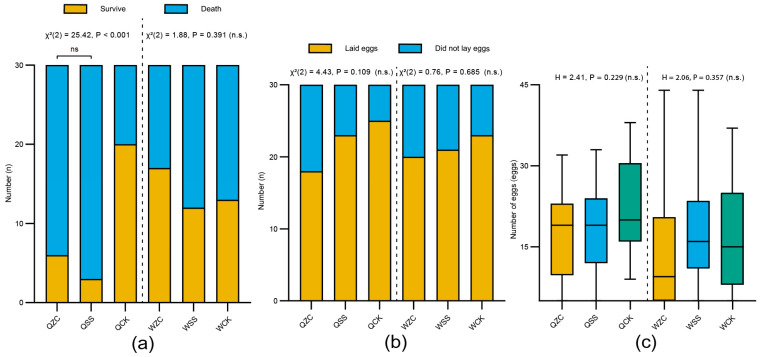
Effects of the removal of the spermatheca on *B. terrestris* females’ overwintering and oviposition. Q = queen; W = worker; ZC = spermatheca-removal treatment; SS = sham-operated treatment; CK = untreated control. (**a**) Effects of different treatments on overwintering survival rate of bumblebee females. Queens were subjected to overwintering for 3 months, whereas workers underwent a 15-day overwintering treatment (QZC: n = 30, QSS: n = 30, QCK: n = 30; WZC: n = 30, WSS: n = 30, WCK: n = 30). (**b**) Effects of different treatments on oviposition rate (QZC: n = 30, QSS: n = 30, QCK: n = 30; WZC: n = 30, WSS: n = 30, WCK: n = 30). (**c**) Effects of different treatments on the number of eggs laid (QZC: n = 18, QSS: n = 23, QCK: n = 25; WZC: n = 20, WSS: n = 21, WCK: n = 23). Box plots consist of the box denoting the interquartile range (IQR), bound by the 25th and 75th percentiles, the median line shown within the box, and the whiskers representing the rest of the data distribution with outliers denoted by points greater than ± 1.5× IQR. Data in (**a**,**b**) were analyzed using the Chi-square test. Data in (**c**) were analyzed using Kruskal–Wallis H test.

After 3 months, there was a highly significant difference in the survival rates of queens among the different treatment groups (QZC = 6/30 (20%), QSS = 3/30 (10%), QCK = 20/30 (66.7); *χ^2^*(2) = 25.42, *p* < 0.001; Figure 4a). Specifically, the survival rate of overwintering queens in the control group was significantly higher than that of both the spermatheca-removal and sham-operated groups, while there was no significant difference between the spermatheca-removal and sham-operated groups (*χ^2^*(1) = 0.52, *p* = 0.47). However, after 15 days of overwintering, there was no significant difference in the survival rates of workers among the treatment groups (WZC = 17/30 (56.7%), WSS = 12/30 (40%), WCK = 13/30 (43.3%); *χ^2^*(2) = 1.88, *p* = 0.391; Figure 4a). The power analysis showed that the probability of a Type II error could not be ruled out (power = 0.214).

### 3.5. Effects of the Removal of the Spermatheca on Female’s Oviposition

In the oviposition experiment, the results showed that neither queens nor workers exhibited significant differences in ovipositional rates among the treatment groups (queen: QZC = 18/30 (60%), QSS = 23/30 (76.7%), QCK = 25/30 (83.3%); *χ^2^*(2) = 4.43, *p* = 0.109; worker: WZC = 20/30 (66.7%), WSS = 21/30 (70%), WCK = 23/30 (76.7%); *χ^2^*(2) = 0.76, *p* = 0.685; Figure 4b). The power analysis showed that the probability of a Type II error cannot be ruled out in either case (power = 0.454 for queens; power = 0.111 for workers).

Prior to statistical analysis, the normality of egg-laying data was assessed using the Shapiro–Wilk test. The results showed that not all groups conformed to a normal distribution. Among the queen groups, QZC (*W* = 0.9, *p* = 0.057) and QSS (*W* = 0.965, *p* = 0.581) were normally distributed, but QCK significantly deviated from normality (*W* = 0.91, *p* = 0.031). Similarly, among worker groups, while WSS (*W* = 0.889, *p* = 0.603) and WCK (*W* = 0.964, *p* = 0.189) passed the normality test, WZC did not (*W* = 0.941, *p* = 0.039). Given the violation of the normality assumption in certain groups, we employed the non-parametric Kruskal–Wallis H test to compare the number of eggs laid among treatment groups. The number of eggs laid by queens and workers within five days after the onset of oviposition showed that there were no significant differences among the treatment groups (queen: QZC = 15.89 ± 9.56 eggs, QSS = 17.78 ± 8.73 eggs, QCK = 21.96 ± 8.23 eggs (mean ± SD); *H* = 2.41, *p* = 0.229; worker: WZC = 13.40 ± 11.38 eggs, WSS = 17.43 ± 10.77 eggs, WCK = 15.91 ± 10.73 eggs (mean ± SD); *H* = 2.06, *p* = 0.357; Figure 4c). These results indicate that the removal of their spermatheca had no significant effect on either the ovipositional capacity or the number of queens and workers. A power analysis showed that the probability of a Type II error was sufficiently low (power > 0.99).

## 4. Discussion

A microsurgical technique for removing the spermatheca of bumblebee females without harming them was developed specifically in this study. The practicality and feasibility of this technique were confirmed by examining its effects on the longevity and reproductive traits of bumblebee females after their spermathecae were removed. We provide a new technique for spermathecal removal, which will facilitate more in-depth studies on the physiological functions of insect organs. For the study of insects, the surgical removal of specific organs has been employed as a crucial method for validating their physiological functions [4,5]. As early as 1975, Villavaso used an insect pin to remove the spermatheca of the boll weevil [22]. The survival rate of individuals after the operation was relatively low, which might be due to the unsuitability of the surgical instrument. In contrast, the high survival rate of individuals in this study may be attributed to the bumblebee-specific scalpel we developed (Chinese Patent No. ZL202411634155.0) [20]. This scalpel was designed based on the anatomical structure and size of the bumblebee spermatheca. A small amount of hemolymph flowed out after surgery using this scalpel, the wound was about 0.34 mm and rapidly formed a scab, which was similar to the reparative process of organ observed in other insects [23], reflecting the strong regenerative capacity of bumblebees; this might be one of the key factors contributing to the high survival rate after the surgery. In addition, the high success rate of this technique is closely related to the unique anatomical features of the bumblebee reproductive system. In contrast, the membrane of the sting chamber in honeybees is opaque, and the diameter of the spermatheca is approximately 1 mm and is encased within a complex tracheal network [24]; it is difficult to identify the spermatheca visually and even more challenging to perform a precise removal. Therefore, this technique is currently not applicable to removing the spermatheca of honeybees. Our study indicates when performing similar removal surgeries on different insect species, it is essential to fully consider their anatomical features and develop specialized instruments. This not only helps to increase the success rate of the surgery, but also provides a guarantee for achieving high survival rates and the accuracy of functional verification.

The spermatheca, as a crucial organ for storing sperm [25], is necessary for sperm acquisition, which occurs through mating. In honeybees, the spermatheca gradually forms a micro-environment that is more suitable for the long-term storage of sperm as the queen reaches sexual maturity [26,27,28,29], which suggests that it might also play a certain role during the mating process. However, our study found that the removal of the spermatheca in both queens and workers did not affect their mating behavior. This result is consistent with the findings of Villavaso [22] in the boll weevil (*Anthonomus grandis*). These findings indicate that the spermatheca is not a necessary prerequisite for mating behavior.

It is worth noting that in this study, the surgical removal of the spermatheca and the sham operation significantly reduced the overwintering survival rate of queens. However, there was no significant difference between the removal surgery and sham-operated groups of the queens. This reduction in the survival rate of queens in overwintering may be attributed to the use of CO_2_ anesthesia in the treated groups, rather than the absence of the spermatheca itself. Previous studies have also shown that CO_2_ exposure can affect the insect metabolic rate, immune function, and endocrine systems, which may consequently reduce survival ability in extreme environments [30,31,32,33]. Boris et al. [34] also reported that the survival rate of queens treated with carbon dioxide during the overwinter stage decreased significantly, which was consistent with this study. However, after the worker group only underwent 15 days of overwintering treatment, this short duration likely did not significantly affect their survival rate. This might be because the overwintering period was too short to have a noticeable effect on their longevity. Additionally, we also conducted CO_2_ anesthesia treatment on both mated and unmated queens of *Bombus terrestris*. The results showed that anesthesia significantly reduced the survival rate of the queens, further supporting the conclusion that CO_2_ exposure negatively impacts overwintering longevity (unpublished data).

Although the spermatheca plays a crucial role in receiving, storing, releasing, and maintaining sperm viability [10], our study showed that bumblebee females are still capable of laying unfertilized eggs after the spermatheca is removed [35,36]. This finding might suggest that the formation of unfertilized eggs and the fertilization process are regulated physiologically through relatively independent pathways. The development and release of eggs mainly depend on the maturity of the ovaries and the levels of hormones [35,36], rather than on the presence of the spermatheca. This phenomenon is particularly common in social insects. For example, although workers of honeybees and ants have degenerated spermathecae and have lost the ability to produce female offspring, they can still produce unfertilized eggs [37,38], and yet they maintain high fitness in social behaviors and longevity [39,40]. By removing the spermatheca, we preliminarily verified that there was no significant effect on the longevity or reproduction-related traits of either queens or workers. This finding provides a foundation for studying whether the spermatheca is functionally highly specialized and for elucidating the mechanisms of its degeneration in workers. This finding suggests that its primary function lies in sperm storage and release, and its absence does not interfere with other reproductive behaviors. This finding lays an experimental foundation for further investigation into the potential physiological roles of the spermatheca beyond sperm storage.

## 5. Conclusions

In this study, we developed, for the first time, a microsurgical technique for removing the spermatheca of bumblebee females, overcoming a major technical barrier to removing reproductive organs in social insects. Using a specially designed scalpel, the spermatheca was successfully removed with minimal wounding. The wounds healed rapidly, and the survival rates of the treatment bees were high, which confirmed the safety and practicality of this technique. The results demonstrated that the removal of the spermatheca did not significantly affect their key physiological traits such as longevity, mating behavior, oviposition capacity, or overwintering capacity in either queens or workers. This finding further validated the feasibility and practicality of this technique. The development of this technique provides a technical foundation for exploring whether the spermatheca has functions beyond sperm storage in social insects and lays the groundwork for investigating the evolutionary mechanisms of spermatheca degeneration in non-reproductive individuals such as workers. Overall, this study represents a major methodological breakthrough and offers novel approaches and experimental tools for studying the functional evolution of reproductive organs in social insects.

## Data Availability

The original contributions presented in this study are included in the article/Supplementary Materials. Further inquiries can be directed to the corresponding author.

## Supplementary Materials

The following supporting information can be downloaded at https://www.mdpi.com/article/10.3390/insects16070734/s1, Video S1: The procedure for removing the spermatheca of bumblebees.

## References

[1] Sullivan J.P., Jassim O., Fahrbach S.E., Robinson G.E. (2000). Juvenile hormone paces behavioral development in the adult worker honey bee. Horm. Behavior..

[2] Sullivan J.P., Fahrbach S.E., Harrison J.F., Capaldi E.A., Fewell J.H., Robinson G.E. (2003). Juvenile hormone and division of labor in honey bee colonies: Effects of allatectomy on flight behavior and metabolism. J. Exp. Biol..

[3] Schroeder T.B., Houghtaling J., Wilts B.D., Mayer M. (2018). It‘s not a bug, it‘s a feature: Functional materials in insects. Adv. Mater..

[4] Strauß J. (2017). The scolopidial accessory organs and Nebenorgans in orthopteroid insects: Comparative neuroanatomy, mechanosensory function, and evolutionary origin. Arthropod Struct. Dev..

[5] Koyama T., Saeed U., Rewitz K., Halberg K.V. (2025). The Integrative Physiology of Hormone Signaling: Insights from Insect models. Physiology.

[6] Kopec S. (1922). Studies on the necessity of the brain for the inception of insect metamorphosis. Biol. Bull..

[7] Couillaud F. (1986). Influence of sexual organs on corpora allata biosynthetic activity in Locusta migratoria. Physiol. Entomol..

[8] Franco M., Fassler R., Goldberg T.S., Chole H., Herz Y., Woodard S.H., Reichmann D., Bloch G. (2023). Substances in the mandibular glands mediate queen effects on larval development and colony organization in an annual bumble bee. Proc. Natl. Acad. Sci. USA.

[9] Ahmed I., Gillott C. (1981). The spermatheca of Melanoplus sanguinipes (Fabr.). I. Morphology, histology, and histochemistry. Int. J. Invertebr. Reprod..

[10] Krueger S., Martins de S., e Silva J., Santos de Oliveira C., Moritz G. (2022). Investigation of the spermathecal morphology, reproductive strategy and fate of stored spermatozoa in three important thysanopteran species. Sci. Rep..

[11] Pascini T.V., Ramalho-Ortigäo J.M., Martins G.F. (2013). The fine structure of the spermatheca in Anopheles aquasalis (Diptera: Culicidae). Ann. Entomol. Soc. Am..

[12] Winnick C.G., Holwell G.I., Herberstein M.E. (2009). Internal reproductive anatomy of the praying mantid Ciulfina klassi (Mantodea: Liturgusidae). Arthropod Struct. Dev..

[13] Dallai R., Mercati D., Gottardo M., Machida R., Mashimo Y., Beutel R.G. (2012). The fine structure of the female reproductive system of Zorotypus caudelli Karny (Zoraptera). Arthropod Struct. Dev..

[14] da Silva I.B., Costa-Leonardo A.M. (2023). Mating mediates morphophysiological changes in the spermathecae of *Coptotermes gestroi* queens. Entomol. Exp. Appl..

[15] Schoeters E., Billen J. (2000). The importance of the spermathecal duct in bumblebees. J. Insect Physiol..

[16] Adams I., Tariq M. (2024). An Overview of Reproduction in Insects. Asian J. Adv. Agric. Res..

[17] Evans E., Burns I., Spivak M. (2007). Befriending Bumble Bees: A Practical Guide to Raising Local Bumble Bees.

[18] Zhuang M., Colgan T.J., Guo Y., Zhang Z., Liu F., Xia Z., Dai X., Zhang Z., Li Y., Wang L. (2023). Unexpected worker mating and colony-founding in a superorganism. Nat. Commun..

[19] Zhang H., Zhou Z., Huang J., Yuan X., Ding G., An J. (2018). Queen traits and colony size of four bumblebee species of China. Insectes Sociaux.

[20] Zhuang M., Li J. (2024). A microsurgical Knife and Its Preparation Method for Spermatheca Removal in Bumblebees. Chinese Patent.

[21] Tasei J. (1994). Effect of different narcosis procedures on initiating oviposition of prediapausing Bombus terrestris queens. Entomol. Exp. Appl..

[22] Villavaso E.J. (1975). Functions of the spermathecal muscle of the boll weevil, Anthonomus grandis. J. Insect Physiol..

[23] Krautz R., Arefin B., Theopold U. (2014). Damage signals in the insect immune response. Front. Plant Sci..

[24] Carreck N.L., Andree M., Brent C.S., Cox-Foster D., Dade H.A., Ellis J.D., Hatjina F., Van Englesdorp D. (2013). Standard methods for *Apis mellifera* anatomy and dissection. J. Apic. Res..

[25] Pascini T.V., Martins G.F. (2017). The insect spermatheca: An overview. Zoology.

[26] Verma L.R. (1974). Honeybee spermatozoa and their survival in the queen‘s spermatheca. Bee World.

[27] Rhodes J.W., Lacey M.J., Harden S. (2007). Changes with age in queen honey bee (*Apis mellifera*) head chemical constituents (Hymenoptera: Apidae). Sociobiology.

[28] Baer B., Eubel H., Taylor N.L., O‘Toole N., Millar A.H. (2009). Insights into female sperm storage from the spermathecal fluid proteome of the honeybee Apis mellifera. Genome Biol..

[29] Al-Lawati H., Kamp G., Bienefeld K. (2009). Characteristics of the spermathecal contents of old and young honeybee queens. J. Insect Physiol..

[30] Farooqui T., Farooqui A.A. (2011). Oxidative Stress in Vertebrates and Invertebrates: Molecular Aspects of Cell Signaling.

[31] Gherlenda A.N., Haigh A.M., Moore B.D., Johnson S.N., Riegler M. (2016). Climate change, nutrition and immunity: Effects of elevated CO_2_ and temperature on the immune function of an insect herbivore. J. Insect Physiol..

[32] Amsalem E., Grozinger C.M. (2017). Evaluating the molecular, physiological and behavioral impacts of CO_2_ narcosis in bumble bees (*Bombus impatiens*). J. Insect Physiol..

[33] Treanore E.D., Amsalem E. (2022). Examining the individual and additive effects of cold storage and CO_2_ narcosis on queen survival and reproduction in bumble bees. J. Insect Physiol..

[34] Baer B., Schmid-Hempel P. (2000). The artificial insemination of bumblebee queens. Insectes Sociaux.

[35] Santos C.G., Humann F.C., Hartfelder K. (2019). Juvenile hormone signaling in insect oogenesis. Curr. Opin. Insect Sci..

[36] Lenaerts C., Monjon E., Van Lommel J., Verbakel L., Vanden Broeck J. (2019). Peptides in insect oogenesis. Curr. Opin. Insect Sci..

[37] Bourke A.F. (1988). Worker reproduction in the higher eusocial Hymenoptera. Q. Rev. Biol..

[38] Gotoh A., Billen J., Hashim R., Ito F. (2016). Degeneration patterns of the worker spermatheca during morphogenesis in ants (Hymenoptera: Formicidae). Evol. Dev..

[39] Strassmann J.E., Queller D.C. (2007). Insect societies as divided organisms: The complexities of purpose and cross-purpose. Proc. Natl. Acad. Sci. USA.

[40] Ronai I., Vergoz V., Oldroyd B. (2016). The mechanistic, genetic, and evolutionary basis of worker sterility in the social Hymenoptera. Advances in the Study of Behavior.

